# Deep Learning Based Multiresponse Optimization Methodology for Dual-Axis MEMS Accelerometer

**DOI:** 10.3390/mi14040817

**Published:** 2023-04-04

**Authors:** Fahad A. Mattoo, Tahir Nawaz, Muhammad Mubasher Saleem, Umar Shahbaz Khan, Amir Hamza

**Affiliations:** 1 Department of Mechatronics Engineering, National University of Sciences and Technology (NUST), Islamabad 44000, Pakistan; 2 National Centre of Robotics and Automation, Islamabad 44000, Pakistan

**Keywords:** deep neural network, dual-axis MEMS accelerometer, microelectromechanical systems (MEMS), multiresponse optimization, deep learning (DL), neural network

## Abstract

This paper presents a deep neural network (DNN) based design optimization methodology for dual-axis microelectromechanical systems (MEMS) capacitive accelerometer. The proposed methodology considers the geometric design parameters and operating conditions of the MEMS accelerometer as input parameters and allows to analyze the effect of the individual design parameters on the output responses of the sensor using a single model. Moreover, a DNN-based model allows to simultaneously optimize the multiple output responses of the MEMS accelerometers in an efficient manner. The efficiency of the proposed DNN-based optimization model is compared with the design of the computer experiments (DACE) based multiresponse optimization methodology presented in the Literature, which showed a better performance in terms of two output performance metrics, i.e., mean absolute error (MAE) and root mean squared error (RMSE).

## 1. Introduction

MEMS inertial sensors, including accelerometers and gyroscopes, are commonly used in various applications such as motion sensing, navigation systems, vibration monitoring, and structural health monitoring [1,2,3,4]. The small size, low power consumption, and low cost of these micromachined sensors make these sensors an excellent alternative to the traditional macroscale inertial sensors. The function of MEMS accelerometers is generally based on different transduction principles such as electrostatic [5,6], piezoelectric [7], piezoresistive [8], and optical [9]. Among these, capacitive MEMS accelerometers are most widely used for different applications, owing to their relatively high dynamic range, small size, and low cost [10].

In the development cycle of MEMS in general and MEMS inertial sensors in particular, it is important to analyze and predict the effect of the geometric design parameters, microfabrication process constraints, and device operating conditions on the output performance characteristics of the sensor. The optimization of MEMS accelerometers is generally carried out by changing one design parameter at a time and estimating its effect on an output response using mathematical models, finite-element-method (FEM) simulations, or topology optimization approaches [11,12,13,14,15]. The capacitive MEMS accelerometers are multiphysics devices which involve a coupled field electro-structural-thermal interaction, and the output performance characteristics of these sensors generally have a contradicting dependence on geometric design parameters and operating conditions. This requires a comprehensive design optimization methodology that allows the MEMS designer to optimize the output performance characteristics of MEMS sensors simultaneously with respect to the device geometric parameters and operating conditions. The design of computer experiments and machine-learning-based optimization techniques have been presented in the Literature for the multi-response optimization of MEMS accelerometers [16,17]. In [17], a machine-learning-based method was proposed for optimization of such parameters based on training a separate model for each output response. However, the solution was not generalizable and had a higher complexity due to the need for training as many models as the number of output responses. It would instead be desirable to have a single unified generic model that enables simultaneous prediction of all the output responses in an efficient manner.

Recently, the use of deep-learning-based approaches have shown highly encouraging results for such combinatorial optimization problems in other fields. However, their use and adoption in the MEMS field is still at its infancy. Deep learning is a subset of machine learning that uses neural networks with multiple layers to learn complex patterns in data [18,19]. It has gained popularity due to its ability to effectively and efficiently learn from large amounts of data and solve complex problems that were previously considered unsolvable [20,21].

To this end, this paper proposes an end-to-end deep-neural-network-based methodology that is aimed at optimization of design parameters by relying on a unified framework that does not require the learning of multiple separate models, and leads to an efficient simultaneous prediction of the accelerometer output characteristics. Indeed, the proposed method allows analysis of the effect of the geometric design parameters and operating conditions on the output performance characteristics of a capacitive MEMS accelerometer in an effective manner. 

## 2. MEMS Accelerometer Design

**Figure 1:**
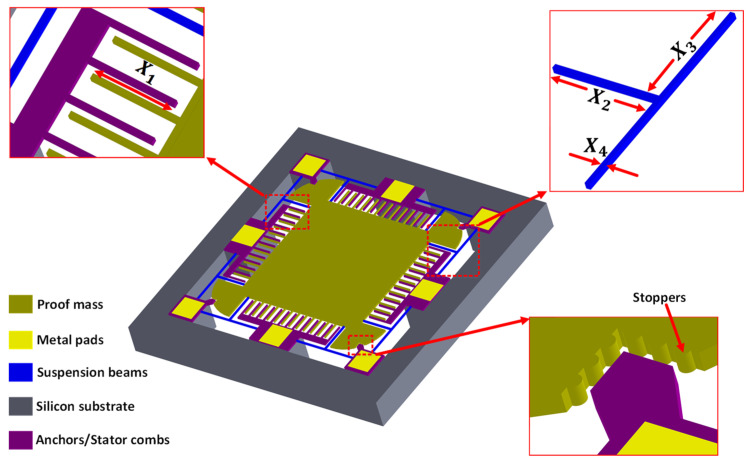
The 2-DoF MEMS capacitive MEMS accelerometer design [17].

The MEMS accelerometer design considered for the implementation of the proposed deep-neural-network-based optimization methodology is shown in Figure 1. The MEMS accelerometer design allows to measure input acceleration in two in-plane axes, thus making it a 2-DoF design. The design consists of a central proof mass with capacitive electrodes attached on the four sides. The T-shaped mechanical suspension beams attached on the four corners of the central proof mass allows to measure input acceleration in both *x*-axis and *y*-axis, while minimizing the cross-axis coupling. For an input acceleration in any axis, the proof mass displaces, and this displacement is measured by using stator and rotor capacitive combs attached on the sides of the proof mass. The stator and rotor combs are arranged in a gap-antigap configuration with a minimum gap value of 2.5 µm, which is defined as per the microfabrication process constraints of the multi-project-wafer-based SOIMUMPs process offered by MEMSCAP Inc., USA [22]. The displacement in the proof mass, corresponding to an input acceleration, results in an air gap change between the stator and rotor combs which leads to a net capacitance change. This net capacitance change is used as an output metric for the measurement of an input acceleration. The dynamic response of the MEMS accelerometer is strongly dependent on the mechanical compliance and stiffness of the suspension beams, which is defined by the geometric dimensions of the beams. Similarly, the net capacitance change for an input acceleration is strongly dependent on the initial air gap between the stator and rotor combs. In addition to the geometric design parameters, the performance of the MEMS accelerometer is affected by the operating temperature and air pressure conditions, which has been discussed in detail in [6,23]. In this work, we have considered both the MEMS accelerometer geometric design parameters and operating conditions as parameters for the optimization. 

The output characteristics considered for simultaneous optimization of the MEMS accelerometer are the central proof mass displacement for an input acceleration, the natural frequency, the pull-in voltage between the stator and rotor combs, the change in the capacitance between the stator and rotor combs for an input acceleration, and Brownian noise equivalent acceleration (BNEA).

## 3. Basics of Deep Learning Model

This section is aimed to develop some background of deep learning for a reader to facilitate an easier understanding of the proposed framework (Section 4) that is built on using these concepts.

The elementary unit of a deep learning network architecture is called a perceptron or an artificial neuron cell. When multiple perceptrons are combined, they form a complex logical system which is referred as a neural network. The simplest form of a perceptron is equivalent to an equation of a line. For the equation for a single perceptron unit, the slope of line (m) is replaced with weights (W), input (x) is replaced with input (X), y-intercept (c) with bias (B), and output (y) is replaced with function of input (f(X)), as shown in Equation (1) [24].

(1) y=mx+c⇒f(X)=WX+B

(2) y=g(f(X))

**Figure 2:**
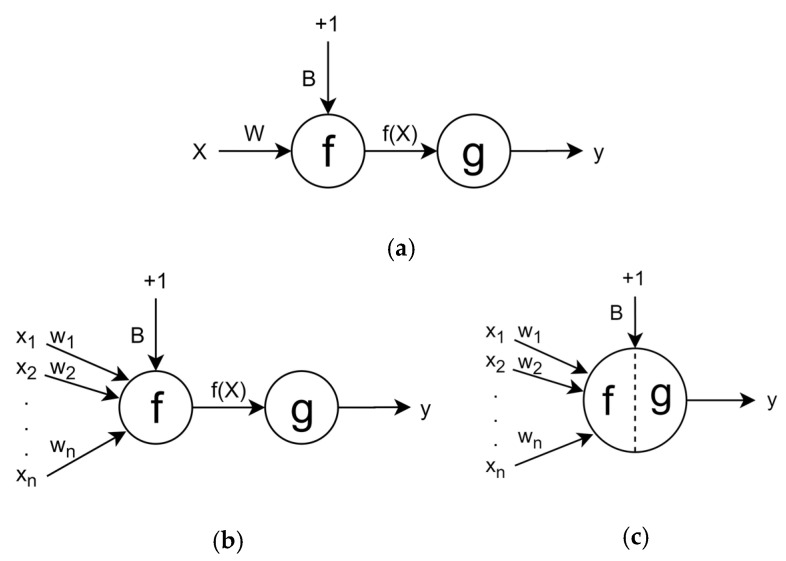
(**a**) Perceptron with single value input, (**b**) perceptron with multi-value input, and (**c**) merging perceptron and activation function.

The output response f(X) is passed through an activation function to add non-linearity in the perceptron unit to make it able to separate data that is not separable by straight lines. In Equation (2), g represents the activation function that is applied to f(X) to obtain the final output response y. Figure 2a shows the schematic representation of a perceptron with single value input. Multi variable input is represented as x_1_, x_2_,…, x_n_, where each x corresponds to an input variable, and each has a corresponding weight (w_1_, w_2_,…, w_n_), as shown in Figure 2b. For a simpler notation, the perceptron and activation function can be presented as a combined unit, as illustrated in Figure 2c.

**Figure 3:**
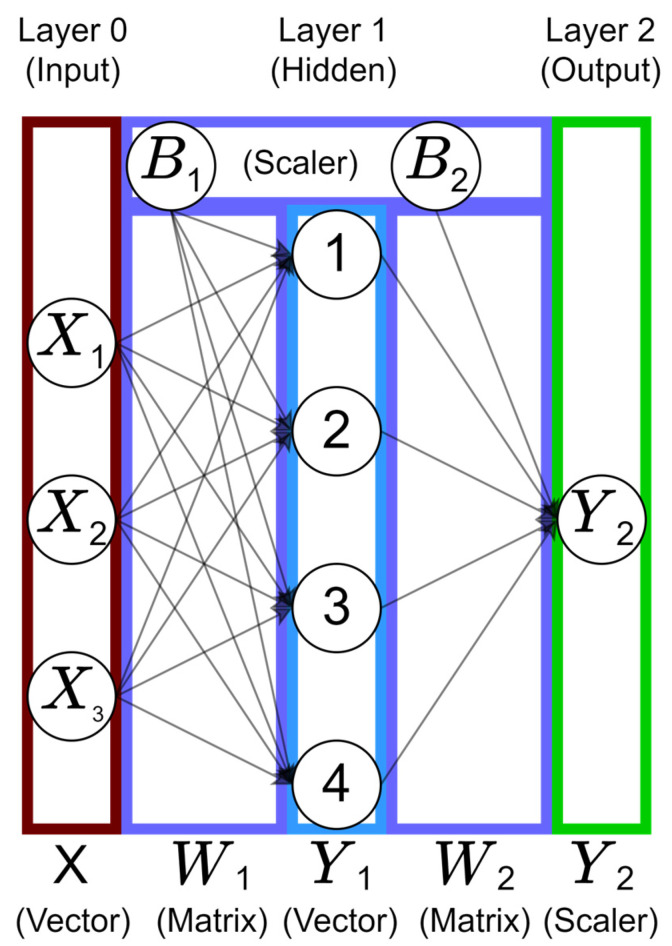
Representation of a neural network containing three inputs and a single output.

Multiple perceptrons can be stacked in the vertical direction to form a layer (e.g., see Layer 1 in Figure 3). Each connection in Figure 3 has a corresponding weight, and the weights are stored in a 2D matrix and represented as W. The final output of this neural network is calculated using Equation (3). The left part of this equation represents the calculation occurring between Layer 0 (input) and Layer 1; first the dot product between input (X) and weight ($W_{1}$) is taken and bias ($B_{1}$) is added to the product. This submission is passed through an activation function (g) to obtain the output for each perceptron in Layer 1 which is represented as $Y_{1}$. The output of Layer 1 acts as the input to the next layer, Layer 2 (output). The calculation between Layer 1 and Layer 2 (output) is the right part of Equation (3), $W_{2}$ and $B_{2}$ are the weights and bias for Layer 2, $Y_{1}$ act as the input for this layer, and $Y_{2}$ is the final output of the neural network.

(3) $$Y_{1}=g\left(W_{1}X+B_{1}\right),\;\;Y_{2}=g\left(W_{2}Y_{1}+B_{2}\right)$$

Such a combination of perceptrons is collectively called a neural network (NN) [25]. Furthermore, a NN can be divided into three parts, an input layer (Layer 0), hidden layer (Layer 1), and an output layer (Layer 2), as shown in Figure 3. When there are 2 or more hidden layers, the NN is called a deep neural network. Since, on its own, a perceptron is simply an equation of a straight line (linear solution), an activation function is therefore needed to introduce non-linearity into the perceptron. Examples of some available activation functions include Sigmoid, Tanh, Rectified Linear Units (ReLU), Exponential Linear Unit (ELU), Swish, and Mish [26]. The learning process of a NN is based on a backpropagation algorithm [27] which uses gradient methods for decreasing the output error. After each training step, the output error is calculated using the prediction made by that current state of the NN. Back propagation is used to calculate the error for each neuron by going through the layers of NN in the reverse direction. Based on this calculated error for each neuron, the weights of the NN are updated. In summary, back propagation updates the weights of the NN to minimize the output error. To improve the generalization ability of NN, hyperparameter tuning [28] is required. A gradient-based algorithm [29] may get stuck at a local minima instead of reaching the global minima, or might even diverge instead of converging to a minima. Hence there is a requirement to select an appropriate combination of activation function, learning rate, number of epochs, batch size, and weight initializer, along with the number of hidden layers and number of perceptrons per layer.

For the multiphysics design optimization of the MEMS accelerometer, we have created a deep neural network that is composed of 4 hidden layers such that each layer has a certain number of perceptrons along with their corresponding activation function for that layer. The number of layers and the number of perceptrons are set empirically. The selection of an activation function depends on the problem at hand. For the hidden layers, we experimented with different activation functions and obtained the best results with ELU in the first hidden layer, and ReLU in the preceding three hidden layers, Equations (5) and (6), respectively. Both ELU and ReLU affect the negative values. ELU uses an exponent operator for its function while ReLU sets the negative value to zero. In Equations (4)–(6), x is the value obtained after the dot product between the input and the weights and the addition of bias to this product.

(4) g(x)=x

(5) $$g\left(x\right)=\{\begin{array}{c} \;\;\;x,\;x>0\\ \;\;\;0,\;x\leq 0 \end{array}$$

(6) $$g\left(x\right)=\{\begin{array}{r} \;\;\;\;x,\;x>0\\ \;\exp\left(x\right)-1,\;x\leq 0 \end{array}$$

The linear activation function is used in the last layer because the optimization of the MEMS accelerometer can be considered a regression problem in which the prediction of continuous values is desired.

## 4. Proposed Deep-Neural-Network-Based Framework

### 4.1. Design, Response, and Desirability Value Details

Table 1 shows the design variables (x_1_, x_2_,…, x_8_) considered for the multiphysics design optimization of the MEMS accelerometer. These design parameters are the geometric parameters and the MEMS accelerometer operating conditions. The significance of the low and high levels for the design parameters has been discussed in [17]. The output responses considered for the optimization are natural frequency (y_1_), proof mass displacement (y_2_), pull-in voltage value (y_3_), capacitance change corresponding to the input acceleration (y_4_), and Brownian noise equivalent acceleration (BNEA) (y_5_). 

### 4.2. General Working of the Proposed Optimization Framework

**Figure 4:**
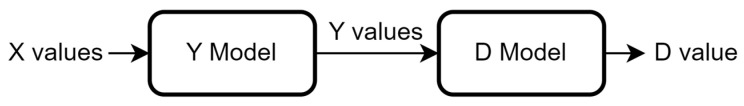
General working of the proposed parameter optimization methodology for the MEMS accelerometer. It is based on calculating the desirability (D) value for optimization using a cascade of two Deep Neural Networks (i.e., Y model and D model). X values correspond to (x_1_, x_2_,…, x_8_); Y values correspond to (y_1_, y_2_,…, y_5_); and D value refers to the desirability value.

The proposed optimization framework for the MEMS accelerometer is based on using a cascade of two separate neural network models, each relying on the architecture as discussed in the previous section. The first model, referred to as the Y model, is designed to predict the output response characteristics of the MEMS accelerometer (y_1_, y_2_,…, y_5_) using the input design parameters (x_1_, x_2_,…, x_8_). The second model is implemented for the simultaneous optimization of the output characteristics of the MEMS accelerometer with respect to the input design parameters, and is referred to as the D model. While the Y model enables a simultaneous prediction of the five output characteristics of the MEMS accelerometer, it does not allow to simultaneously optimize these five output characteristics with respect to the design parameters. The simultaneous optimization of the output characteristics is achieved through the D model, which is based on maximizing the desirability function corresponding to the optimization objective function [25,30]. Based on the output of the D model, the values of the eight input design parameters are ranked and the combination which gives the maximum desirability values is presented as the optimized solution. Figure 4 provides a high-level pictorial overview of the working of the proposed framework.

### 4.3. Output Response Prediction Model

**Figure 5:**
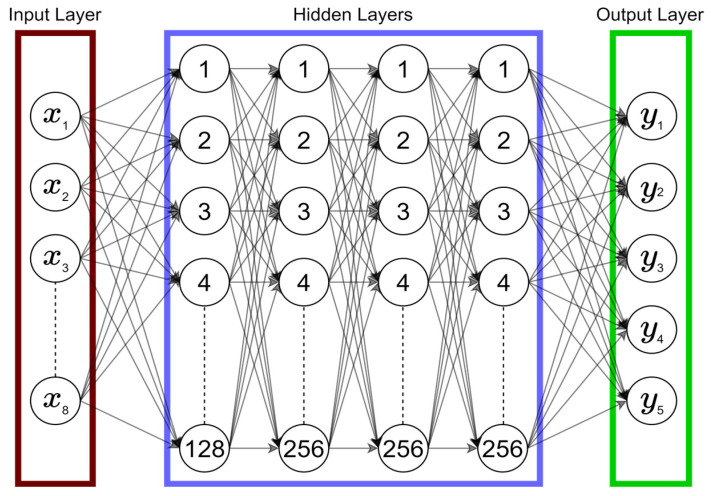
Representation of the architecture of the Y model.

Figure 5 represents the deep neural network that is used to train the Y model for the output responses prediction. The input layer contains input features (x_1_, x_2_,…, x_8_) corresponding to design variables and the output layer contains the corresponding output responses (y_1_, y_2_,…, y_5_) that are to be predicted.

**Figure 6:**
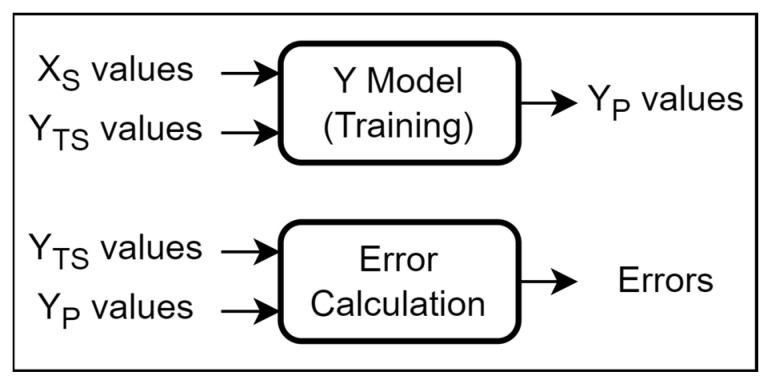
Steps for the training process and the evaluation of the Y model.

To train a model for predicting Y_P_ values for a set of X values, we used the data provided by [17]. The data has 80 rows of values, each row has a set of X values generated using Latin hypercube sampling and will be represented as X_S_; Ref. [17] obtained the Y values after performing simulations and these values will are represented as Y_TS_. For our work we have normalized the values between 0 and 1 to standardize the scale of each input and output value. A split of 80/20 was made for hyper-parameter tuning and training of the model. Here, the assumption is that the simulated data (as provided by [17]) used for training the Y model was generated taking into account the realistic design conditions of the MEMS accelerometer. Figure 6 shows the steps involved in the training process as well as the evaluation of the Y model.

In comparison to [17], where five separate individual models were trained to obtain each output response value, the proposed method is based on training a single model to obtain all the output response values. The Y model is evaluated according to two error metrics, which are mean absolute error (MAE) and root mean squared error (RMSE), calculated using Equations (7) and (8), respectively.

(7) $$MAE=\frac{1}{k}\overset{k}{\underset{i=1}{\sum }}\left|y_{oi}-y_{pi}\right|$$

(8) $$RMSE=\sqrt{\frac{1}{k}\overset{k}{\underset{i=1}{\sum }}{\left(y_{oi}-y_{pi}\right)}^{2}}$$

where $y_{oi}$ is the true output value at index i and $y_{pi}$ is the corresponding prediction value, and k is the total number of samples. The errors obtained are compared with the error values of [17]. It is observed that the proposed Y model has consistently outperformed [17] as shown in Table 2.

### 4.4. Effect of Design Parameters on the Output Responses

The effect of variation of each design parameter (x_1_, x_2_,…, x_8_) on the output responses (y_1_, y_2_,…, y_5_) is also observed to obtain a deeper insight into their respective behaviors. In this regard, each input parameter is varied across its range while keeping all of the remaining input parameters fixed at the average of the low and high levels, as defined in Table 1. Since each output response has a different range and unit, they are normalized between 0 and 1 for comparisons.

**Figure 7:**
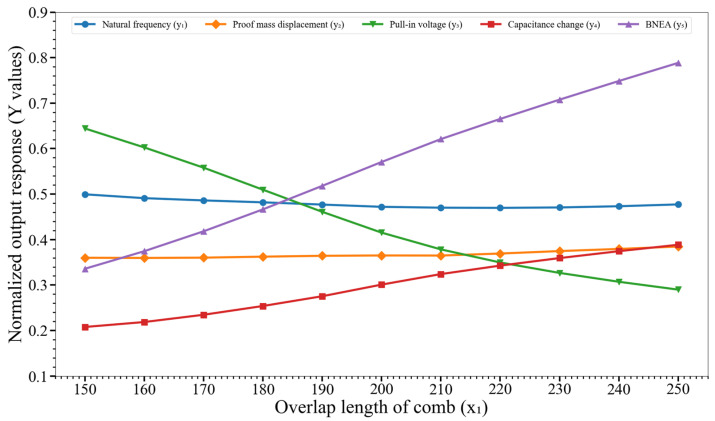
Effect of variation of the overlap length of comb (x_1_) on the output responses y_1_, y_2_,…, y_5_. Key. y_1_: natural frequency; y_2_: proof mass displacement; y_3_: pull-in voltage; y_4_: capacitance change; and y_5_: BNEA.

Figure 7 shows the effect of variation of the overlap length of comb (x_1_) on y_1_, y_2_,…, y_5_. The graphs show that there is a much stronger impact of the variation of x_1_ on the pull-in voltage (y_3_) and BNEA (y_5_) than on the natural frequency (y_1_), proof mass displacement (y_2_), and capacitance change (y_3_). The results show that with an increase in the x_1_, the pull-in voltage value decreases and BNEA increases for the MEMS accelerometer.

**Figure 8:**
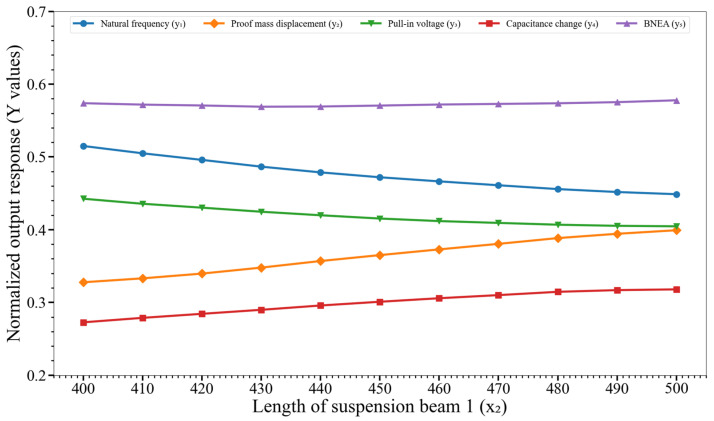
Effect of variation of the length of the suspension beam 1 (x_2_) on the output responses y_1_, y_2_,…, y_5_. Key. y_1_: natural frequency; y_2_: proof mass displacement; y_3_: pull-in voltage; y_4_: capacitance change; and y_5_: BNEA.

Figure 8 shows the effect of variation of the length of the suspension beam 1 (x_2_) on y_1_, y_2_,…, y_5_. The results show that with an increase in the x_2_ value, the natural frequency and pull-in voltage value for the MEMS accelerometer decreases while the proof mass displacement and capacitance change for an input acceleration increases. Moreover, the effect of change in the x_2_ on the MEMS accelerometer BNEA value is negligible. 

**Figure 9:**
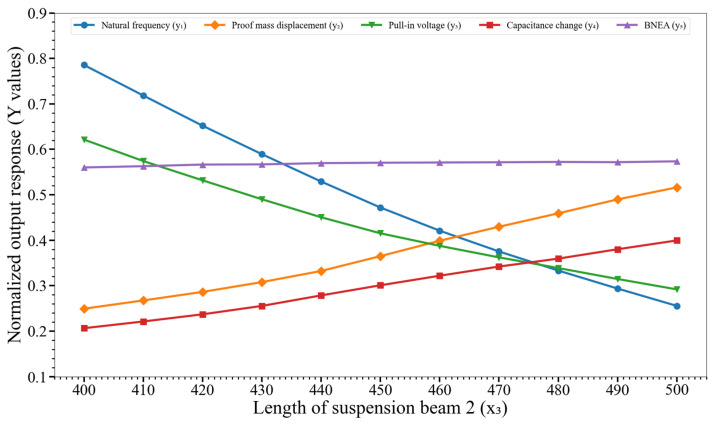
Effect of variation of the length of suspension beam 2 (x_3_) on the output responses y_1_, y_2_,…, y_5_. Key. y_1_: natural frequency; y_2_: proof mass displacement; y_3_: pull-in voltage; y_4_: capacitance change; and y_5_: BNEA.

Figure 9 shows the effect of the variation of the length of suspension beam 2 (x_3_) on y_1_, y_2_,…, y_5_. The strongest effect of the variation of x_3_ is clearly visible on the natural frequency (y_1_) that matches with the findings of [17]. Additionally, the graph also shows that x_3_ also contributes at varying levels towards the proof mass displacement (y_2_), pull-in voltage (y_3_), and capacitance change (y_4_).

**Figure 10:**
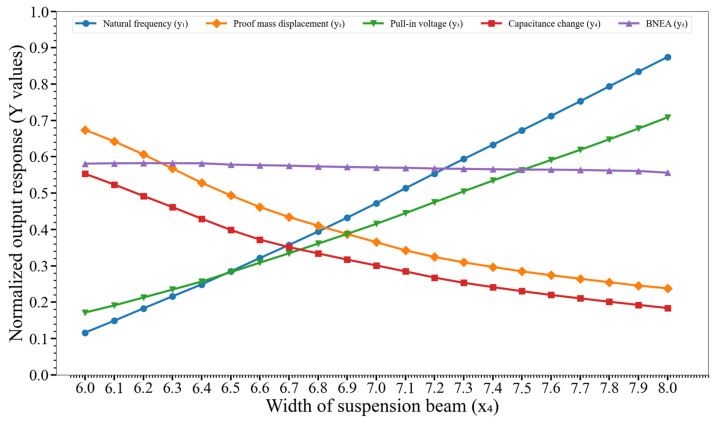
Effect of variation of the width of the suspension beam (x_4_) on the output responses y_1_, y_2_,…, y_5_. Key. y_1_: natural frequency; y_2_: proof mass displacement; y_3_: pull-in voltage; y_4_: capacitance change; and y_5_: BNEA.

Figure 10 shows the effect of variation of the width of the suspension beam (x_4_) on y_1_, y_2_,…, y_5_. The graph shows that there is a significant impact of variation of x_4_ on the natural frequency (y_1_), proof mass displacement (y_2_), pull-in voltage (y_3_), and capacitance change (y_4_).

**Figure 11:**
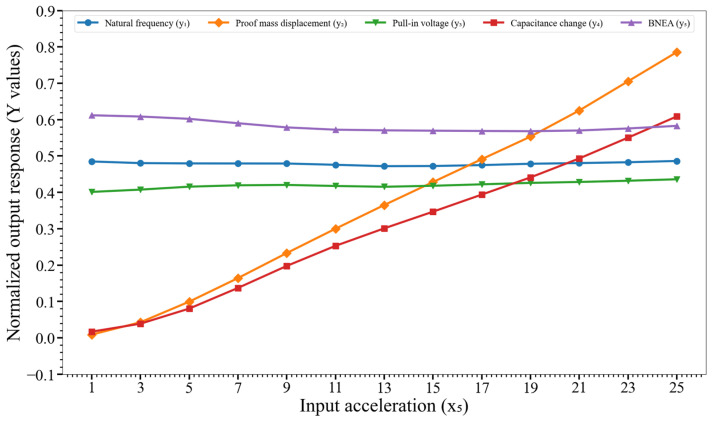
Effect of variation of the input acceleration (x_5_) on the output responses y_1_, y_2_,…, y_5_. Key. y_1_: natural frequency; y_2_: proof mass displacement; y_3_: pull-in voltage; y_4_: capacitance change; and y_5_: BNEA.

Figure 11 shows the effect of variation of the input acceleration (x_5_) on y_1_, y_2_,…, y_5_. It is evident from the graphs that x_5_ strongly impacts the proof mass displacement (y_2_) and capacitance change (y_4_).

**Figure 12:**
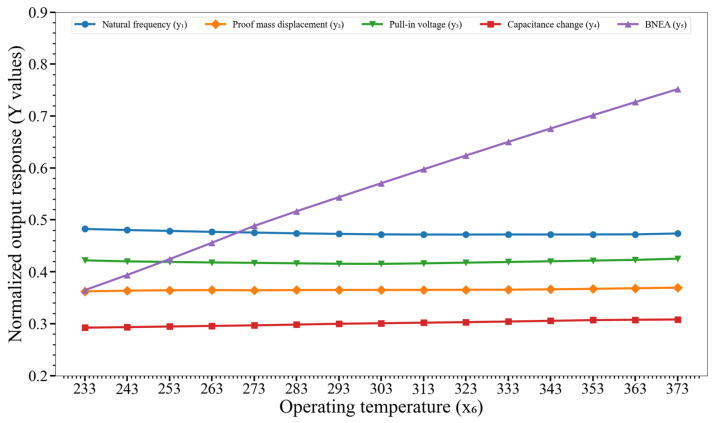
Effect of variation of the operating temperature (x_6_) on the output responses y_1_, y_2_,…, y_5_. Key. y_1_: natural frequency; y_2_: proof mass displacement; y_3_: pull-in voltage; y_4_: capacitance change; and y_5_: BNEA.

Figure 12 shows the effect of variation of the operating temperature (x_6_) on y_1_, y_2_,…, y_5_. The graphs that only BNEA (y_5_) is impacted by the variation of x_6_, whereas the remaining output responses are not perturbed. 

**Figure 13:**
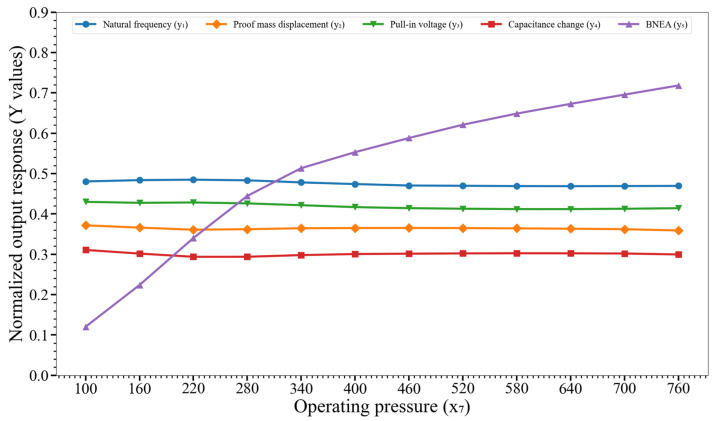
Effect of variation of the operating pressure (x_7_) on the output responses y_1_, y_2_,…, y_5_. Key. y_1_: natural frequency; y_2_: proof mass displacement; y_3_: pull-in voltage; y_4_: capacitance change; and y_5_: BNEA.

Figure 13 shows the effect of variation of the operating pressure (x_7_) on y_1_, y_2_,…, y_5_. The behavior is similar to that observed for the case of x_6_, i.e., x_7_ also impacts only the BNEA (y_5_), without really perturbing the remaining output responses.

**Figure 14:**
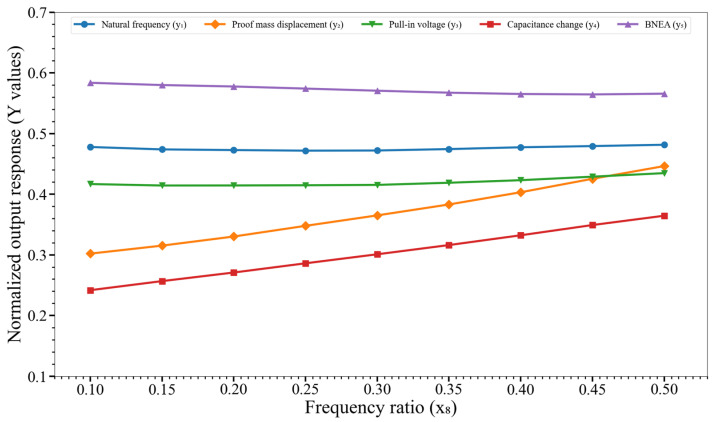
Effect of variation of the frequency ratio (x_8_) on the output responses y_1_, y_2_,…, y_5_. Key. y_1_: natural frequency; y_2_: proof mass displacement; y_3_: pull-in voltage; y_4_: capacitance change; and y_5_: BNEA.

Figure 14 shows the effect of variation of the frequency ratio (x_8_) on y_1_, y_2_,…, y_5_. There is not a strong impact of variation of x_8_ on any output response that is in line with observations of [17]; though y_2_ and y_4_ appear slightly perturbed.

The results presented in Figure 6, Figure 7, Figure 8, Figure 9, Figure 10, Figure 11, Figure 12 and Figure 13 allow to analyze the effect of the design parameters of the MEMS accelerometer on the five output responses simultaneously. The sensitivity analysis for each design parameter has been performed in terms of the effect of the variation of the design parameters on the output responses, and then comparing them with the results presented in [17], which showed a consistent behavior for each design parameter. Thus, the proposed deep-learning-based Y model allows to efficiently explore the MEMS accelerometer design space. Additionally, the effectiveness of the model has already been quantitatively demonstrated in the form of the comparison of the predicted output responses (y_1_, y_2_,…, y_5_) obtained using the proposed Y model with those obtained using the method in [17], based on MAE and RMSE scores (Table 2). Moreover, unlike the procedure adopted in [17] for generating the simulated data that was extremely time consuming (taking extended periods of time to complete), the proposed Y model (once trained) offers an alternative for generating more data (where needed) in the design space in an accurate and time-efficient manner without the need to perform simulations over longer periods of time. In fact, in the next section, the trained Y model is used to generate a larger dataset as required for training the D model.

## 5. Multiresponse Optimization Using D Prediction Model

### 5.1. Training of the D Prediction Model

**Figure 15:**
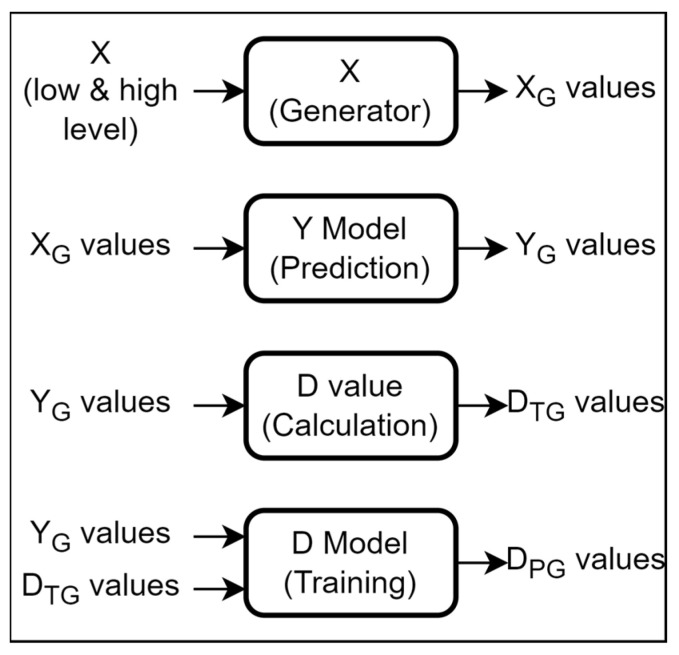
Steps involved in the training process of the D Model.

For the training of the D model, we use a larger dataset generated using the Y model. The deep neural network used for training the D model is exactly the same (except, of course, the input and output layers) as the one for the Y model (Figure 5). As for the dataset, it has been generated by first creating a list of different combinations of X inputs. This was performed by incrementing from the low and high level of X values. The increment was set between 15 and 25 percent of the low level, which generated a set of X values, referred to as X_G_ values (Figure 15). The X_G_ values are passed to the Y model to obtain the corresponding Y_G_ values. For each Y_G_ value, the D_G_ value was estimated using the same approach used in [17], and this obtained D value is represented as the D_TG_ value, or true value of desirability for the generated set of X_G_ values. A total of 3125 rows of values were obtained. All the values were normalized between 0 and 1 to standardize the scale of each input and output value. A split of 80/20 was made for hyperparameter tuning and training of the model. For training of the D model, the input layer therefore contains y_1_, y_2_,…, y_5_ and the output layer contains only a desirability value. The manual formula-based calculation of D (as in [17]) is thus replaced with a robust deep-learning-based D model.

### 5.2. Multi-Response Optimization

**Figure 16:**
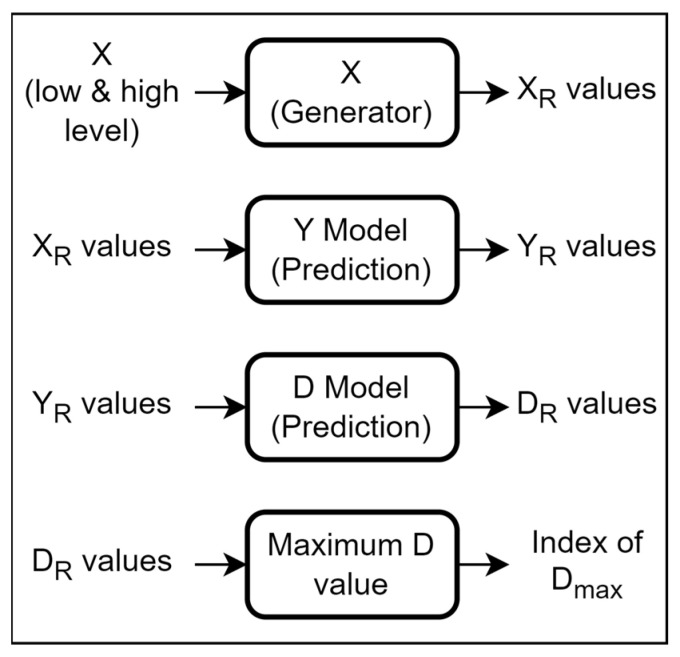
Final Optimization pipeline.

To find the optimized values for X with respect to maximum D, we have proposed a method as illustrated in Figure 16. For the optimization process a dataset of about 100 K values was generated for the X values with an increment value below 10 percent of the lower bound; this set of X values is represented as X_R_. The X_R_ is fed through the Y model to obtain the Y_R_. The obtained Y_R_ values are fed to the D model to obtain the D_R_, which are the D values for the corresponding design parameters. Then, the index of the maximum D value is searched, and the corresponding Y and X to this maximum index are considered as the optimized x_1_, x_2_,…, x_8_ values. Table 3 presents the values obtained from the proposed method and the values reported in [17].

To further validate the obtained results, we performed the statistical significance testing at the standard 5% significance level using the two-sample *t*-test. The Y values (y_1_, y_2_,…, y_5_) predicted using the optimized X values (x_1_, x_2_,…, x_8_) based on the proposed method are listed in Table 3. For comparison, we computed the Y values (referred to as observed Y values) by performing FEM simulations in CoventorWare^®^ software (Coventor, Raleigh, NC, USA) using the same X values as obtained based on the proposed method. The computed observed Y values are as follows: *y*_1_ = 3096.43 Hz, *y*_2_ = 0.676 μm, *y*_3_ = 7.0303 V, *y*_4_ = 521 fF, and *y*_5_ = 0.7959 μg/$\sqrt{\mathrm{Hz}}$. Here, the *t*-test is performed to test the null hypothesis that the data in the two samples (predicted Y values and observed Y values) is derived from independent random samples having normal distributions of equal means and equal but unknown variances. The results of the *t*-test show that the null hypothesis is not rejected with *p*-value = 0.98, thus confirming that the data in the two samples is statistically highly similar.

## 6. Conclusions

This paper proposed a design optimization methodology for a dual-axis microelectromechanical systems (MEMS) capacitive accelerometer based on the use of a cascade of two deep neural network (DNN) models. Each model is made up of 4 hidden layers. The first hidden layer is composed of 128 perceptrons and ELU as activation function. The other three layers have 256 perceptrons and ReLU as activation function. A linear activation function was used in the output layer, as a regression system is required. The first instance is named as Y model and is used to predict the output response values. Y Model was trained on the original 80 values, as made available by [17]. Using the trained Y model and the ranges of design parameters, a larger dataset of 3125 values was generated. This generated dataset was used to train the second instance that is named as the D model. The output of the D model is the desirability value on which the design parameters are ranked and accordingly optimized.

The proposed method enabled an analysis of the effect of the individual design parameters on the output responses of the sensor using Y model. Additionally, the D model allowed a simultaneous optimization of the multiple output responses of the MEMS accelerometers in an efficient manner. Compared to the work [17] in which five separate models based on the Gaussian process were trained (one for each output response), plus the use of a desirability function, the proposed method is computationally less complex and more efficient as it offers a unified solution using a DNN model (replacing five separate models of [17]), which has been demonstrated to be more accurate and effective as compared to [17]. The results of the proposed method are also validated by means of statistical significance testing.

## Figures and Tables

**Table 1:** Design parameters for the MEMS device.

Notation	Design Parameters	Low Level	High Level
x_1_	Overlap length of comb	150 μm	250 μm
x_2_	Length of suspension beam 1	400 μm	500 μm
x_3_	Length of suspension beam 2	500 μm	500 μm
x_4_	Width of suspension beam	6 μm	8 μm
x_5_	Input acceleration	1 g	25 g
x_6_	Operating temperature	233.15 K	373.15 K
x_7_	Operating pressure	100 Torr	760 Torr
x_8_	Frequency ratio	0.1	0.5

**Table 2:** Comparison of the predicted output responses (y_1_, y_2_,…, y_5_) obtained using the proposed Y model with those obtained using the method in [17], in terms of MAE and RMSE.

Output Response	MAE		RMSE	
	Proposed	[17]	Proposed	[17]
Natural frequency (y_1_)	12.67 Hz	29.64 Hz	15.41 Hz	41.19 Hz
Proof mass displacement (y_2_)	0.004 μm	0.024 μm	0.004 μm	0.034 μm
Pull-in voltage (y_3_)	0.065 V	0.085 V	0.072 V	0.134 V
Capacitance change (y_4_)	5.292 fF	10.179 fF	6.28 fF	14.05 fF
BNEA (y_5_)	$0.004\;\mu g/\sqrt{\mathrm{Hz}}$	$0.019\;\mu g/\sqrt{\mathrm{Hz}}$	$0.005\;\mu g/\sqrt{\mathrm{Hz}}$	$0.029\;\mu g/\sqrt{\mathrm{Hz}}$

**Table 3:** Comparison of the obtained optimized X values (x_1_, x_2_,…, x_8_) using the proposed method with those reported in [17]. The corresponding Y values (y_1_, y_2_,…, y_5_) are also listed.

Optimized Values Reported in [17]								
	*x*_1_	*x*_2_	*x*_3_	*x*_4_	*x*_5_	*x*_6_	*x*_7_	*x*_8_
Design Parameters (X values)	153.6 μm	403.6 μm	500 μm	6.26 μm	25 g	300 K	760 Torr	0.50
	***y*** **_1_**	***y*** **_2_**	***y*** **_3_**	***y*** **_4_**	***y*** **_5_**			
Output Responses (Y values)	3036.4 Hz	0.903 μm	6.76 V	676.2 fF	$0.81\;\mu g/\sqrt{\mathrm{Hz}}$			
**Optimized Values Obtained Using the Proposed Method.**								
	***x*** **_1_**	***x*** **_2_**	***x*** **_3_**	***x*** **_4_**	***x*** **_5_**	***x*** **_6_**	***x*** **_7_**	***x*** **_8_**
Design Parameters (X values)	150.0 μm	430.0 μm	500 μm	6.40 μm	25 g	300 K	760 Torr	0.45
	***y*** **_1_**	***y*** **_2_**	***y*** **_3_**	***y*** **_4_**	***y*** **_5_**			
Output Responses (Y values)	3160.0 Hz	0.723 μm	7.22 V	571.0 fF	$0.83\;\mu g/\sqrt{\mathrm{Hz}}$			

## Data Availability

No new data were created or analyzed in this study. Data sharing is not applicable to this article.
